# Google search behavior for meningitis and its vaccines: an infodemiological study

**DOI:** 10.1186/s12883-021-02258-w

**Published:** 2021-06-23

**Authors:** John Angelo Luigi S. Perez, Adrian I. Espiritu, Roland Dominic G. Jamora

**Affiliations:** 1 Institute for Neurosciences, St. Luke’s Medical Center, Quezon City & Global City, Philippines; 2 Department of Clinical Epidemiology, College of Medicine, University of the Philippines Manila, Manila, Philippines; 3 Department of Neurosciences, College of Medicine, Philippine General Hospital, University of the Philippines Manila, Manila, Philippines

**Keywords:** Infodemiology, Meningitis, Vaccines, Google Trends™

## Abstract

**Background:**

The internet has made significant contributions towards health education. Analyzing the pattern of online behavior regarding meningitis and vaccinations may be worthwhile. It is hypothesized that the online search patterns in meningitis are correlated with its number of cases and the search patterns of its related vaccines.

**Methods:**

This was an infodemiological study that determined the relationship among online search interest in meningitis, its worldwide number of cases and its associated vaccines. Using Google Trends™ Search Volume Indices (SVIs), we evaluated the search queries “meningitis,” “pneumococcal vaccine,” “BCG vaccine,” “meningococcal vaccine” and “influenza vaccine” in January 2021, covering January 2008 to December 2020. Spearman rank correlation was used to determine correlations between these queries.

**Results:**

The worldwide search interest in meningitis from 2008 to 2020 showed an average SVI of 46 ± 8.8. The most searched topics were symptoms, vaccines, and infectious agents with SVIs of 100, 52, and 39, respectively. The top three countries with the highest search interest were Ghana, Kazakhstan, and Kenya. There were weak, but statistically significant correlations between meningitis and the BCG (*ρ* = 0.369, *p* < 0.001) and meningococcal (*ρ* = 0.183, *p* < 0.05) vaccines. There were no statistically significant associations between the number of cases, influenza vaccine, and pneumococcal vaccine.

**Conclusion:**

The relationships among the Google SVIs for meningitis and its related vaccines and number of cases data were inconsistent and remained unclear. Future infodemiological studies may expand their scopes to social media, semantics, and big data for more robust conclusions.

## Introduction

Meningitis is a neurologic emergency that is associated with significant morbidity and mortality. It is the inflammation of the meninges of the brain and spinal cord and may have various etiologies such as infectious, autoimmune, paraneoplastic and even drug-related [1]. It has several risk factors such as human immunodeficiency virus infection, malnutrition, overcrowding, and incomplete vaccinations. The majority of mortalities are from infectious meningitis and bacterial meningitis can become rapidly fatal and survivors become severely disabled [1]

Immunization is well-known to be one of the preventive measures against meningitis [1]. Vaccines targeting *Hemophilus influenzae* type b, *Neisseria meningitidis*, and *Streptococcus pneumoniae* have significantly decreased the number of cases of meningitis worldwide [1]. According to the World Health Organization (WHO), vaccinations have reduced the incidence of meningitis in Africa by as much as 58% and the risk of epidemics has decreased by 60% [2]. One of the pioneering vaccines developed in collaboration with the WHO, UNICEF, and the Bill & Melinda Gates Foundation have reduced the incidence of meningitis by 94% after six months in areas with successful vaccination drives [3]. Despite vaccination efforts, meningitis remains a global problem, especially in developing countries and the contiguous African Meningitis Belt [1].

The internet has made a significant contribution towards health education and health-seeking behavior with online news and social media being the most popular sources of information [4, 5]. In a systematic review, publications utilizing information and communication technologies including the internet generally fell into five categories: a) evaluation of health-related content, b) development and evaluation of health services, c) development and evaluation of health literacy tools, d) interventions to improve health literacy, and e) to promote online health information-seeking behavior [6].

In the case of vaccinations, the internet has significantly affected the volume and quality of information available and in some cases became either facilitator or barrier of health information [7]. Since vaccinations are one of the most important preventive measures against meningitis, analyzing the pattern of online behavior regarding meningitis and vaccinations may be worthwhile. It is hypothesized that the online search patterns in meningitis are correlated with its number of cases and the search patterns of its related vaccines.

This field of studying internet behavior in health is a relatively new concept that may be covered by the field of infodemiology [8]. It is defined as the science of distribution and determinants of information in an electronic medium, specifically the Internet, or in a population, with the ultimate aim to inform public health and public policy [8]. One of the most accessible methods of studying online search patterns is the use of Google Trends™ (https://trends.google.com). It is a free and publicly available online portal that provides data on Google search queries. It can provide geospatial and temporal patterns on approximately 3 billion search queries daily [9].

The purpose of this study was to determine the associations among online search interest in meningitis, its worldwide number of cases and its associated vaccines. The scope of this study involved vaccine-preventable etiologies of meningitis such as *influenza*, *tuberculosis*, *meningococcus*, and *streptococcus*.

## Methods

Online interest was measured as search volume index (SVI) provided by the Google Trends™. SVI is a proprietary index that estimates search activity by taking the proportion of searches of interest relative to the most searched term within a specified geography and timeframe [10]. The SVIs are normalized to geography and time and are then assigned a value from 0 to 100.

Google Trends™ was accessed by visiting http://trends.google.com and a combined worldwide search query evaluation for “meningitis”, “pneumococcal vaccine”, “BCG vaccine”, “meningococcal vaccine”, and “influenza vaccine” in January 2021. The search was limited from January 2008 to December 2020 and search queries as topics were used instead of freehand keywords. SVI data was then returned by Google Trends™ and downloaded as Microsoft Excel spreadsheets, version 2101 (Redmond, WA: Microsoft Corp.). The global case numbers of meningitis from 1990 to 2019 were obtained from the Global Health Data Exchange Database (GHDx) as comma-separated values [11]. The figures were generated using Microsoft Word, version 2101.

The non-parametric Spearman rank correlation was used to determine correlations between the SVIs of meningitis, the SVIs of vaccines, and the worldwide number of cases of meningitis. A Durbin-Watson test for autocorrelation was used to determine any seasonality in SVI. All data were using IBM SPSS Statistics for Windows, Version 26.0 (Armonk, NY: IBM Corp). The p-value was set at 0.05.

## Results

### General search interest trends

**Fig. 1:**
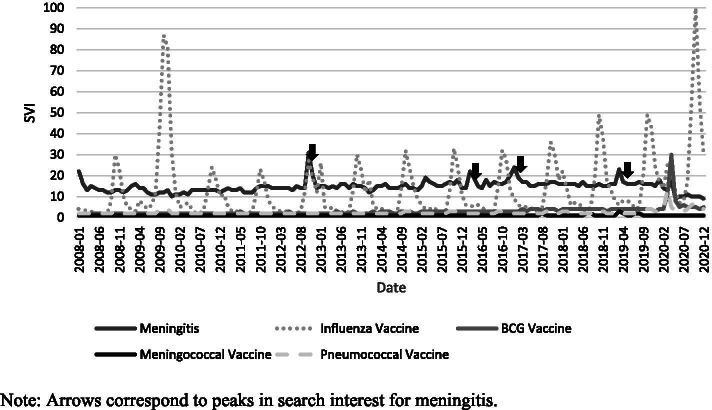
Search interests for combined queries on meningitis and its vaccines from 2008 to 2020

Worldwide search interest in meningitis from 2008 to 2020 showed an average SVI of 46 ± 8.8. The most related topics searched were symptoms, vaccines, and infectious agents with SVIs of 100, 52, and 39, respectively. The top five countries with the highest search interest were Ghana, Kazakhstan, Kenya, Paraguay, and Italy (see Table 1). Overall, the trend in search interest for meningitis increased over time with an abrupt drop in May 2020 (see Fig. 1). There were several spikes in SVI in October 2012, February 2016, January 2017, and March 2019.

**Table 1:** Top 50 countries with the highest search volume indices from 2008-2020 and their total cases up to 2019

Country	SVI	Number of cases	Country	SVI	Number of cases
1. Ghana	100	656,602	26. Costa Rica	44	1,193
2. Kazakhstan	88	148,066	27. Finland	44	1,625
3. Kenya	84	1,469,781	28. Ecuador	42	3,439
4. Paraguay	82	69,311	29. Netherlands	42	6,425
5. Italy	80	92,267	30. Australia	42	9,830
6. Chile	73	57,306	31. Dominican Republic	42	5,557
7. South Africa	73	571,477	32. Argentina	42	14,001
8. Slovenia	71	9,501	33. France	40	24,651
9. Ireland	68	18,993	34. Sweden	40	2,687
10. Brazil	66	160,512	35. Hungary	40	2,813
11. Norway	66	28,609	36. Colombia	40	11,644
12. United Kingdom	64	33,162	37. Algeria	40	21,625
13. New Zealand	60	1,717	38. Portugal	40	6,607
14. United States	57	26,781	39. Ukraine	40	32,485
15. Indonesia	57	150,591	40. Switzerland	40	2,142
16. Denmark	53	3,020	41. Romania	40	5,438
17. Bolivia	51	2,657	42. Austria	37	2,414
18. Jordan	51	9,454	43. Germany	37	20,571
19. Russia	51	76,676	44. Czech Republic	37	3,790
20. Philippines	51	85,124	45. Greece	35	3,537
21. Nigeria	48	619,661	46. Slovakia	35	2,244
22. Belarus	46	6,482	47. Venezuela	35	9,131
23. Belgium	44	3,780	48. Egypt	33	60,577
24. Spain	44	17,813	49. Saudi Arabia	33	10,756
25. Canada	44	4,510	50. Morocco	33	24,546

A search query combining meningitis and its associated vaccines showed generally low search volumes for pneumococcal, meningococcal, and BCG vaccines (see Fig. 1). Search volumes for influenza vaccine were cyclical due to the seasonal nature of the disease. A Durbin-Watson test showed a coefficient of 0.598, indicating positive autocorrelation [12].

### The global number of cases of meningitis

**Fig. 2:**
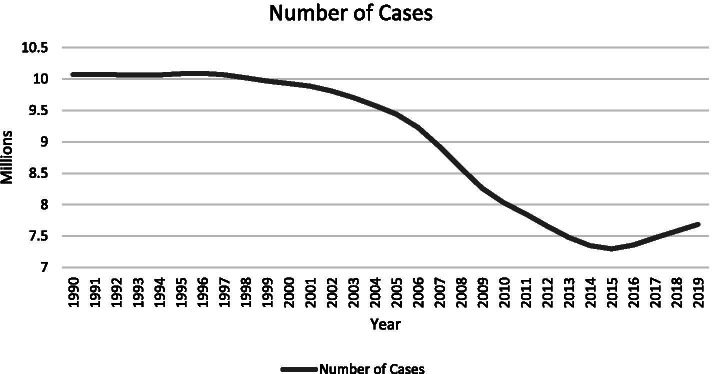
Number of meningitis cases globally from 1990–2019 according to the global burden of disease database [6]

According to the GHDx Database, the number of meningitis cases worldwide as of 2019 was 7,683,539 (10%). The top three countries with the highest cases were India (n = 2,099,361, 15%), Nigeria (n = 619,660, 21%), and Pakistan (n = 401,311, 19%). There was an overall decline in the number of cases starting around 1997. However, the cases increased again in 2016 (see Fig. 2).

The top searches by country, queries, and rising queries each for meningitis and its vaccines are shown in Table 2. The majority of searches for “meningitis” came from developing countries while a mix of developed and developing countries was searching for its vaccines. The search queries for meningitis were mostly signs and symptoms, while vaccine queries were mostly direct searches. All searches were multi-lingual and had queries in English, Japanese, Italian, Russian, and Greek.

**Table 2:** Results of search volume analysis and search queries for terms "meningitis," "influenza vaccine," "BCG vaccine," "pneumoccocal vaccine" and "meningoccocal vaccine"

**Search Term**	**Country**		**Top Queries**		**Rising Queries**	
Meningitis	Ghana Kazakhstan Paraguay Kenya Italy	100 93 89 83 79	Meningitis Meningite Symptoms meningitis Viral meningitis Bacterial meningitis	100 27 12 7 6	Vacina meningite b Suntik meningitis Vaksin meningitis Gejala meningitis мeнингит этo	Breakout
Influenza Vaccine	Japan Canada USA Finland Taiwan	100 77 71 67 62	Flu Flu shot Flu vaccine インフルエンザ インフルエンザ 予防 接種	100 53 21 19 16	Flu shot 2020 Flu shot near me インフルエンザ 予防 接種 Flu shot 2019 Flu vaccine 2020	Breakout
BCG Vaccine	Japan Kazakhstan Nepal Taiwan Ukraine	100 98 97 94 92	BCG BCG vaccine бцж BCG vacuna Vacina BCG	100 20 11 7 7	コロナ コロナ BCG BCG COVID бцж этo BCG Corona	Breakout
Pneumococcal Vaccine	Austria Portugal Czechia Taiwan Finland	100 78 72 69 68	Pneumonia Prevenar Pneumococcal Pneumonia vaccine Pneumococcal vaccine	100 91 82 67 48	Prevenar 13 Prevnar 13 How often pneumonia shot Pneumo 13 Prevenar 13 vacuna	Breakout
Meningococcal Vaccine	Australia Brazil Greece Portugal USA	100 88 84 67 62	Meningitis Meningitis vaccine Meningococcal Meningococcal vaccine Vacina	100 94 74 59 42	Vacina meningite b Vacina acwy Meningo b Vacina meningite acwy Meningo b vacina	Breakout

### Associations among online search interest in meningitis, its worldwide number of cases and its associated vaccines 

There were weak, but statistically significant correlations between meningitis and the BCG and meningococcal vaccines (see Table 3). There was no statistically significant correlation with the number of cases of meningitis.

**Table 3:** Spearman rank correlations between the SVI of meningitis, its related vaccinations, and worldwide number of cases

Variable	Spearman rank coefficient	*P*-value
Pneumococcal vaccine	0.011	0.895
Influenza vaccine	0.051	0.526
BCG vaccine	0.369	< 0.001*
Meningococcal vaccine	0.183	0.022*
Worldwide number of cases	0.354	0.179

*SVI* Search volume index

## Discussion

Our study shows that people who search for meningitis on Google looked for definitions of the disease and its symptoms. The majority of searches came from developing countries. This was consistent with the literature that affirms that the morbidity and mortality burden of meningitis are greater in resource-poor nations [11]. As for the vaccine keywords, the majority searched for general information on the vaccine, the nearest place to get them, and their frequency of administration. Interestingly, most of the searches came from developed countries. This may be due to the lower disease burden of meningitis as shown by their case numbers. Moreover, these nations are already working on prevention rather than acute infection. The pattern of keyword searching that certain countries employ may possibly give public health workers an indication on the current phase of a region in the epidemic stage spectrum. In our study, the countries battling ongoing epidemics are searching for definitions, signs and symptoms, while developed countries are inclined to focus on prevention and are searching for vaccine information, timing and location.

The number of cases of meningitis worldwide showed a generally decreasing trend (see Fig. 2). However, online search interest was slowly increasing (see Fig. 1). This can be due to multiple factors such as increasing awareness of the disease, expanding internet access, and the rising importance of prevention. An exception to this observation was a sudden drop in search interest in May 2020 and this may be due to the coronavirus disease (COVID-19) pandemic dominating search interest globally. The increase in search volumes for influenza, BCG, and pneumococcal vaccines beginning March 2020 were likewise likely related to vaccine interest amidst the COVID-19 pandemic. This underscores again the role of the internet in health-seeking behavior and health education: even if global prevalence decreases, the internet may be used to facilitate and sustain awareness, prevention, and treatment of meningitis. The internet becomes part of the armamentarium of public health both in prevention and management in developed and developing countries.

Google Trends™ not only provides baseline search data over time, but they also provide insight into sudden spikes in search volume. Several studies have similarly used Google Trends™ to establish baseline search data and look for changes over time. For example, infodemiological studies using Google Trends™ on multiple sclerosis [13], status epilepticus [14], and stroke [5] have been performed. In the case of meningitis, there were four notable spikes in search volume throughout the past 12 years. On further investigation, these spikes coincided with news events at those times.

In October 2012, there was an outbreak of fungal meningitis associated with the New England Compounding Center (NECC) in the United States. It was found that the NECC has been compounding injections of steroids contaminated with *Exserohilum rostratum* and *Aspergillus fumigatus* [15].

February 2016 was an eventful month due to overlapping international events. First, there was a large outbreak of pneumococcal and meningococcal meningitis in Ghana [16]. Second, there was likewise a spike of cases in Tuscany, Italy during a meningitis epidemic that has been ongoing since 2015 [17]. At the same time in the United Kingdom, the daughter of Matt Dawson, a British professional football player, died of meningococcal meningitis [18]. This spurred a petition to make meningococcal vaccinations mandatory for children up to 11 years old. The petition gathered 823,349 signatures by its conclusion [19]. Meanwhile in the United States, there was an outbreak among students in Santa Clara University, California and likewise contributed to the interest in vaccination [20]. Unsurprisingly, the top three countries with the highest search interest in this period were Ghana, Italy, and the United Kingdom.

The peak in January 2017 was caused mainly by searches coming from Italy. While this interest was not due to increasing cases, there was a highly publicized shift in the policies of local health authorities that strengthened vaccine coverage and allowed substantial savings up to 50% with the assistance of insurance [21]. This announcement was made on December 29, 2016 and media coverage lasted up to May 2017.

Finally, the most recent spike occurred in March 2019. This was when former Brazilian president Luiz Inacio Lula da Silva, who was imprisoned for corruption allegations, was given temporary leave to attend his grandson’s funeral who died of meningitis [22]. Both the prison furlough and funeral garnered media attention.

These patterns show that traditional media such as television, radio, and newsprint still have a role in disseminating health information. These media can serve as gateways to information and spur further health-seeking behavior at the user’s own time and convenience. In order to have a successful public health information policy, both traditional media and the internet must be used together.

The present analysis showed that while there were statistically significant correlations between meningitis and the BCG and meningococcal vaccines; the relationships appeared weak. It was also unclear if these correlations correspond to the overall decrease in meningitis cases worldwide. There were no correlations seen with the other vaccines. There was clear seasonal interest in the influenza vaccine, but this was not observed in meningitis. Search interests in meningitis, therefore, do not appear to correlate with search interests on its vaccine or its number of cases.

Non-parametric Spearman correlation was chosen as case numbers of meningitis and search interest of the various keywords over the study period were hypothesized to be non-normally distributed. This was supported by this study’s results as shown in Figs. 1 and 2. Meningitis is a chronic global problem with multiple outbreaks and not a point epidemic hence an assumption of normality cannot be made. Similarly, search interest is expected to be non-normal as access to the internet is not uniform throughout the world and the global population is unlikely to be searching for the same topic within the same timeframe. As internet access varies throughout the world and different countries experience outbreaks at different times, the assumption of normality cannot likewise be made.

Testing for autocorrelation is also important since some of the vaccines of interest are seasonal in nature, such as with the influenza vaccine. Inspection of data showed a seasonal trend in the search interest for the influenza vaccine (see Fig. 1) and this is supported by the Durbin-Watson test which showed positive autocorrelation. The Durbin-Watson test is used in time-series analysis and helps differentiate if the seasonal trend is a natural sequential order or is due to some other external cause [12]. In the case of this analysis, the positively autocorrelated search interest in influenza vaccine is an inherent pattern and makes the overall correlation with meningitis more reliable.

Counterintuitively, these results are not entirely unexpected. As previously discussed, the internet may play a role in the sustenance of awareness and search interest in meningitis despite its decreasing numbers worldwide. As for the weak to absent correlations of meningitis with vaccines, it may be possible that the messaging of these vaccines is not clear as they relate to meningitis. Another possibility is that the other complications of tuberculosis and meningococcus such as disseminated TB or meningococcemia are emphasized. This means that meningitis alone may not be a sufficient surrogate measure for vaccine awareness and that other related illnesses must be investigated as well. Meningitis is not the only complication these vaccines prevent. For health workers who will undergo similar studies in the future, search terms must be selected holistically and should reflect the full spectrum of the disease to detect more nuanced correlations.

There were several limitations to this study. First, this study was confined to Google searches and did not measure search interest in social media. While there were spikes in search interest in meningitis, these were attributable to meningitis dominating news headlines. The extent of how specific social media platforms could have contributed to search interest is unknown. The study was also subject to the penetration of internet access as not all countries may have the same ability or extent to perform Google searches. In effect, this study was only able to measure the search behavior of those with internet access and may underrepresent resource-poor countries. Another limitation was that the Global Burden of Disease Database reports all-cause meningitis and does not differentiate among bacterial, viral, fungal, or tuberculous meningitis. Finally, there was a paucity of validated data on Google search behavior and other online behavior such as in social media. It is then difficult to establish true causality and relationships between search terms. The possible explanations discussed in this analysis are hypotheses that warrant further study.

While Google Trends™ results are increasingly being used to provide a general study of online search behavior, future infodemiological studies can focus on social media for a more precise and granular evaluation of internet behavior. Not only will these future studies use search data, but internet crawlers may be programmed to measure interactions and engagement, analyze semantics and even perform big data analytics for improvement of public health and policy.

## Conclusion

The present study found weak correlations between Google SVIs for meningitis and the BCG and meningococcal vaccines without any correlations with worldwide meningitis cases. The majority of online search interest came from the developing countries with queries related to definitions, signs, and symptoms while developed countries searched for vaccine information. Worldwide search interest was increasing despite decreasing numbers of meningitis globally. Despite the weak correlations found, this study serves as a step towards more complex and robust infodemiological studies in the future. Given the relative youth of the field of infodemiology, its methods may still be further refined and its limitations overcome. The internet plays a significant role in medicine hence infodemiology will continue to grow as an emerging tool towards better public health and healthcare.

## Data Availability

The datasets used and/or analyzed during the current study are available from the corresponding author on reasonable request.

## Abbreviations

COVID-19: Coronavirus disease
GHDx: Global Health Data Exchange Database
NECC: New England Compounding Center
SVI: Search volume index
